# Dual Repurposing of End-of-Life BWRO Membranes: Ultrafiltration Membranes for Advanced Wastewater Treatment and Cation Exchange Membranes for Fungal Microbial Fuel Cells

**DOI:** 10.3390/membranes15010005

**Published:** 2024-12-27

**Authors:** Anissa Somrani, Mehri Shabani, Zaineb Mohamed, Kholoud Abohelal, Salam S. Alsharari, Ahmed Hannachi, Noreddine Ghaffour, Maxime Pontié

**Affiliations:** 1 Physics Department, College of Sciences, Jouf University, Sakaka P.O. Box 2014, Saudi Arabia; zmohamed@ju.edu.sa (Z.M.);; 2 ESAIP La Salle, CERADE, 18 Rue du 8 Mai 1945, 49180 Saint-Barthélemy d’Anjou, Cedex, France; 3 Group of Analysis & Processes, Faculty of Sciences, University of Angers, 2 Bd. A. de Lavoisier, 49045 Angers, Cedex 01, France; maxime.pontie@univ-angers.fr; 4 Department of Biology, College of Sciences, Jouf University, Sakaka P.O. Box 2014, Saudi Arabia; 5 Laboratory of Engineering Processes and Industrial Systems, Chemical Engineering Department, National School of Engineers of Gabes, University of Gabes, Street Omar Ibn El Khattab, Gabes 6029, Tunisia; 6 Environmental Science and Engineering Program, Biological and Environmental Science and Engineering (BESE) Division, King Abdullah University of Science and Technology, Thuwal 23955-6900, Saudi Arabia

**Keywords:** BW30-400, Cl2 attack, cation exchange membrane, ultrafiltration, fungal microbial fuel cell, recycling of SWE

## Abstract

The objective of this study is to evaluate the degradation of end-of-life BWRO membranes sourced from a factory in France by analyzing their water permeability, roughness, and chemical composition in order to diagnose the level of degradation incurred during their first life cycle in water softening. Following this, two new applications for the end-of-life BWRO membranes were investigated: (i) as ultrafiltration membranes (UF) for domestic effluent treatment and (ii) as cation exchange membranes (CEM) for use in fungal microbial fuel cells (FMFC). The UF membrane was renovated with an acetic acid treatment and, subsequently, used for domestic effluent filtration. The cation exchange membrane was developed in two steps: (i) chlorine treatment and (ii) the deposition of an Amer Sil layer, a functional coating formed by an interpenetrating polymer network (IPN) made of sulfonated polyether sulfone (S-PES) in a cross-linked matrix of acrylic acid and divinylbenzene.

## 1. Introduction

Over time, due to fouling and chemical degradation, reverse osmosis (RO) membranes become less efficient and need to be replaced. Brackish water reverse osmosis (BWRO) desalinization plants replace about 5% of their membranes each year, while sea water reverse osmosis (SWRO) desalination plants replace up to 20% of their membranes due to the harsher conditions that diminish membrane performance [1]. This translates into over 2 billion end-of life (EoL) reverse osmosis modules being discarded annually in the world, generating more than 32,000 tons of plastic waste [2]. Currently, most of these discarded membranes are disposed of in landfills with some being incinerated for energy recovery [3].

To improve the sustainability of membrane-based desalination technologies, it is crucial to adopt a circular economy approach to RO modules [4]. This approach focuses on reducing resource use, repurposing, recycling, and material recovery to minimize environmental impacts, enhance economic viability, and achieve social equity [5]. This can be achieved by introducing innovative business models that repurpose end-of-life membranes in new efficient ways.

One approach to addressing about 2 million expected end-of-life spiral-wound RO membranes by 2025 and contributing to the circular economy is to repurpose these membranes into other membranes, such as ultrafiltration (UF) [2,6]. By removing the top layer of polyamide (PA), an RO membrane can be transformed into a UF membrane with high permeability, offering excellent performance for various applications [7,8,9]. In 2021, Ahmed et al. [10] demonstrated that this conversion, when applied to gray water, resulted in higher water recovery and the effective removal of *E. coli*. In the same year, Khaless et al. [11] studied the salt rejection of used RO membranes and suggested their re-use in phosphoric acid treatment. Additionally, a life cycle assessment (LCA) and economic analysis reported by various researchers highlighted the environmental benefits of recycling RO membranes, showing positive outcomes and proving that recycled modules could be sold competitively at EUR 80 [2,12,13].

Dagar et al. [14] examined the use of spiral-wound UF combined with RO for treating wastewater from the pulp and paper industry. The study highlighted the importance of pre-treatment in reducing total suspended solids (TSS) by UF, while RO was more effective in removing total dissolved solids (TDS) and organic contaminants, achieving high quality permeate. This approach ensures compliance with water quality standards for industrial reuse. Qu et al. [15] explored the performance of UF membranes under various transmembrane pressures for tertiary wastewater treatment. It noted challenges with membrane fouling due to effluent organic matter and highlighted strategies to optimize the rejection of hydrophobic pharmaceuticals. The study suggested operating in moderate pressure ranges to balance fouling and efficiency. The previous studies [14,15] modeled the operational parameters of spiral-wound UF membranes, focusing on cost-effectiveness and fouling control. They emphasized the role of optimized flushing protocols to maintain efficiency during the treatment of secondary effluent.

One such novel approach proposes giving EoL RO membranes a second life as separators in fungal microbial fuel cells (FMFC) by fully removing their active polyamide layer, thus facilitating indirect recycling [16]. Nafion^®^ is a separator commonly used in chemical fuel cells and microbial electrochemical technologies (MET) to enable proton transfer. Cation exchange membranes (CEMs) are crucial for fuel cell performance. While the Nafion^®^ membrane is the preferred separator for microbial fuel cells (MFCs) [17], it does have some drawbacks, including a tendency to foul and scale and its high cost. Research is exploring alternative materials that can offer similar benefits, such as high proton conductivity and stability, without the limitations of the Nafion^®^ membrane [16,18].

There is much documentation on the tendency for biofouling of Nafion^®^ in MFC applications [17,19,20]. This build-up of biofilm has a negative impact on its performance and, as a result, anti-biofilm techniques have been developed to resolve this, but high cost remains a concern [21]. A lot of research has been devoted to end-of-life RO membranes, with a sixfold increase in studies on this topic between 2000 and 2022, according to data from Science Direct. For example, Somrani et al. [16] developed a new ANIMAX PEM membrane from EoL RO membranes that had been treated with chlorine and had a PSS layer deposited, making it suitable for fungal MFCs.

Research in microbial electrochemical technologies (MET) has demonstrated that composite Nafion^®^ membranes and sulfonated aromatic polymers can be both more cost-effective and more efficient than traditional Nafion^®^ membranes [19,22,23,24]. Various polymers, such as poly(allylamine hydrochloride), poly(vinyl alcohol), poly(phenylene) sulfide, poly(-propylene), polystyrene, cellulose esters, and chitosan have been tested as MFC separators but these have a variety of disadvantages such as fragility, sensitivity to pH changes, aging, poor conduction, and biofouling [25,26,27]. To address this, Singha et al. [25] created a nanocomposite membrane using polybenzimidazole (PBI) to improve conductivity. Further research on transforming EoL RO membranes has shown that treatments like oxidation can eliminate fouling and alter the dense polyamide layer to achieve NF or UF filtration properties [28,29].

This article investigates the potential to convert BWRO membranes into low-pressure UF membranes using acetic acid treatments or into cation exchange membranes (CEM) through chlorine treatment with the deposition of a sulfonated layer to enhance microbial fuel cell performance. First, we examine the primary reasons for membrane degradation, using data from desalination plants to illustrate how fouling and aging occur. Then, we outline chemical processes that may be applied to used RO membranes to repurpose them for use in domestic effluent treatment and microbial fuel cells. Finally, we conduct a life cycle assessment (LCA) to compare acetic acid and chlorine treatments to evaluate which method offers the most sustainable second life for these membranes.

## 2. Materials and Methods

### 2.1. Membrane

The EoL RO membranes used in this study were BW30-400 models from Dow Chemical Company (Midland, MI, USA), sourced from an industrial water softening facility in France. These membranes had experienced significant declines in performance caused by clogging. To investigate the cause, we conducted an autopsy and cut the used BW30 membranes into sections of a relevant size for a UF/NF/RO test setup, with each piece having an effective filtration surface area of 200 cm^2^. Detailed specifications of this membrane are available on Dow Chemical’s website [30]. The old pristine BW30 membrane was an industrial spiral wound membrane (stocked in bisulfite 1% aqueous solution for 10 years) that was not used before; its properties were previously investigated in a study focused on fungal microbial fuel cell applications (see [16]).

### 2.2. Bench-Scale Laboratory Pilot

Experiments were performed using a bench-scale NF/RO laboratory unit from OSMONICS (Osmonics Inc., Minnetonka, MN, USA), which can operate at pressures up to 69 bar. Each membrane sample tested in this high-pressure module had a surface area of 200 cm^2^. During testing, a low flow rate of 5% and a tangential flow velocity of 0.2 m/s were applied, achieving a Reynolds number of 400 [31]. The applied transmembrane pressures ranged from 0 to 20 bars, with the temperature maintained at 20 °C.

The water flow through the membrane was calculated using Equation (1):

*J* = *A* (Δ*P* − *σ*
*Δπ*) (1)

where *J* is the flux (L·h^−1^·m^−2^), *A* is the hydraulic permeability (L·h^−1^·m^−2^·bar^−1^), *σ* is the coefficient of reflection (between 0 and 1), Δ*P* is the transmembrane pressure (bar), and *Δπ* is the transmembrane osmotic pressure (bar).

The osmotic pressure was determined by applying Equation (2):

*Π* = *i*
*c*
*R*
*T* (2)

where *Π* is the osmotic pressure, *i* is the van’t Hoff index, *c* is the molar concentration of solute, *R* is the ideal gas constant, and *T* is the temperature.

### 2.3. ROSA^TM^ Software

This setup allowed us to compare the hydraulic permeability of the end-of-life (EoL) RO fouled membrane, the EoL RO membrane soaked overnight in a chlorine solution (Cl_2_), and the EoL RO membrane soaked overnight in vinegar with the reference membrane (BW30-400).

To simulate membrane performance, we ran ROSA^TM^ software (version 9.1), developed by the membrane manufacturer. In our study, the BW30-400 membrane was used as a reference against which to compare the properties of new membranes. The simulation conditions were as follows: a temperature of 20 °C, well water with a low silt density index (SDI < 3), recovery rates of <20%, and pressure between 2 and 20 bars. Once the relevant data and conditions were entered into the software, we were able to compare the hydraulic permeability of the different studied membranes with the reference membrane (BW30-400).

### 2.4. Membrane Transformation Protocol

#### 2.4.1. Preparation of UF Membranes

The BW30 membrane, previously used for water softening, was treated to repurpose it for ultrafiltration. The EoL RO membrane was immersed in an acetic acid solution (pH 2.5 at 20 °C, with 50 g/L concentration) and soaked overnight at room temperature (around 20 °C) with a stirring speed of 200 rpm (BW30 1 night in vinegar). After soaking, the membranes were rinsed thoroughly with ultra-pure water and stored in plastic containers with ultra-pure water until further testing for domestic effluent treatment applications.

#### 2.4.2. Preparation of CEM Membranes

The end-of-life RO membrane was carefully cut into a 3 cm diameter circular piece to fit the membrane cell. Two steps were taken to modify the membrane sample for use as a proton exchange membrane (PEM). First, the end-of-life RO membrane was immersed in an 11,000 ppm Cl_2_ solution and soaked overnight at room temperature (~20 °C) with a stirring speed of 200 rpm. Following this exposure, the end-of-life RO membranes were thoroughly rinsed with UP water and stored in plastic tubes containing UP water for later use. Chlorine solutions were prepared by diluting commercial Cl_2_ with ultrapure (UP) water. For the end-of-life BW30 membrane transformation, sodium hypochlorite (NaOCl) was diluted in a K_2_HPO_4_/KHPO4 buffer (pH 8.2). The oxidation process followed a protocol established elsewhere [28]. Second, after chlorine treatment, the membrane surface was coated with a cation exchange active layer (Amer Sil deposit), a functional interpenetrating polymer network (IPN) made of sulfonated polyether sulfone (S-PES) in a cross-linked matrix of acrylic acid and divinylbenzene. The prepared membrane, referred to as ANIMAX2, was tested following these modifications: one night of chlorine treatment and one night of polyelectrolyte adsorption from the Amer Sil.

### 2.5. Membrane Characterization

The membranes were examined under a scanning electron microscope (SEM), the Zeiss EVO model (Dublin, CA, USA), which operated at 7 kV to reduce irradiation damage and achieve a resolution of a few nanometers (nm) with a working distance of 13 mm. Observations were conducted at magnifications up to ×5000. To enhance image quality, an ultra-thin carbon coating (2–5 nm) was applied by evaporation in a vacuum [31].

Membrane topography was assessed with a VEECO Nanoscope III atomic force microscope (AFM, Plainview, NY, USA), operated in contact mode in the air at a scan rate of 1 Hz and a resolution of 400 × 400 pixels. Imaging was conducted with Bruker cantilevers (Paris, France) at resonant frequencies between 17 and 20 kHz and spring constants of 0.44 to 0.63 nN. AFM images were analyzed with WSxM 5.0 software, which enables detailed examination of surface features such as average roughness and peak-to-valley ratios. Before imaging, the following treatment was applied [31]: membranes were dried at room temperature in a desiccator and mounted on steel discs using double-sided tape. Imaging focused on small membrane areas (50 μm × 50 μm), and the average roughness (Ra) was calculated as the mean surface point height relative to the central plane using Equation (3).

(3) $$R_{a}=\frac{1}{L_{x}\;L_{y}}\;\int_{0}^{L_{y}}\int_{0}^{L_{x}}\left|z\;(x,y)\right|.dx.dy$$

where *z*(*x*,*y*) is the height from the center plane and *L_x_* and *L_y_* are the dimensions of the analyzed surface plane.

The optical analysis was conducted on the membranes surfaces with a VHX-7000 digital microscope from Keyence SAS company (Paris, France). The mean roughness Ra, which is the mean value of the height of points of the surface relative to the center plane within the defined area, was calculated using Equation (3).

Membrane functional groups (old pristine RO membranes, after Cl_2_ treatment and with Amer Sil deposition) were identified through Fourier transform infrared (FTIR) spectroscopy, spectrum one (Thermo Scientific, Waltham, MA, USA), using 1 cm^2^ membranes samples placed in a Thermo Scientific Nicolet™ iS50 in the Mid-IR measurement range (4000–400 cm^−1^) with a 4 cm^−1^ resolution. The infrared spectra were performed on the old pristine RO membrane, the EoL RO membrane, the EoL RO membrane after one night in chlorine (Cl_2_), and the EoL RO membrane after one night in chlorine (Cl_2_) followed by the Amer Sil deposit.

To determine the crystallographic structure of the fouling materials, XRD measurements were conducted using a Bruker D8 (Cu K*α* at 40 kV) X-ray diffractometer. Scans were performed over the 2*θ* range of 10–100° with the scanning rate of 1°/min and 0.02° MNP Cu K*α* radiation.

### 2.6. Preparation of Artificial Domestic Effluent

Artificial domestic effluent was prepared by mixing clay particles, 3-methyl-4-nitrophenol (MNP), and tap water. Clay was added at 0.2 g per liter of tap water, yielding approximately 50.3 NTU. MNP is an organic micropollutant and a primary by-product of fenitrothion, an organophosphorus insecticide commonly used in the Sahel region to combat desert locust invasions.

### 2.7. Microbial Fuel Cell Experiments

#### 2.7.1. Reagents

Paracetamol (APAP) was obtained from Sigma-Aldrich (St. Louis, MO, USA). A 0.1 M phosphate buffer solution (PBS) at pH 7.4 served as the supporting electrolyte. Methylene blue (MB), para-aminophenol (PAP), APAP, and sulfanilamide (SFA)solutions were prepared using analytical-grade chemicals and deionized water, which had a pH of 6.5, conductivity less than 1 µS/cm, and a total organic carbon (TOC) concentration under 0.1 mg/L, provided by the Elga Lab Water ultrapure-water system (Purelab-UV-UF, Elga, Buckinghamshire, UK).

The carbon paste electrodes (CPE) wereobtained by thoroughly combining 5 mg of cellulose fibers, 30 mg of silicone oil, and 65 mg of graphite powder (analytical grade, ultra F, <325 mesh, from Alfa) in a mortar, as outlined elsewhere [32]. A portion of this composite mixture was then packed into the cylindrical cavity of a Teflon^®^ tube, which was fitted with a copper wire for electrical contact to ensure consistent contact during electrochemical measurements. The surface that would come into contact with the solution was polished on weighing paper to achieve a smooth finish before use.

#### 2.7.2. *Scedosporium dehoogii* Fungal Culture

This study employed the *Scedosporium dehoogii* strain UA 110350859-01, isolated from soil near Angers, France. The strain was maintained on a yeast extract-peptone-dextrose (YEPD) agar medium containing, per liter, 5 g yeast extract, 5 g peptone (Sigma-Aldrich, St. Louis, MA, USA), 20 g dextrose (Sigma-Aldrich, St. Louis, MA, USA), and 0.5 g chloramphenicol (Prolabo, Paris, France). Conidia were collected after a two-week incubation by flooding the agar surface with 15 mL of ultrapure water. The suspension was then filtered through a 40 μm sterile nylon filter, and conidia were pelleted by centrifugation at 4000× *g* for 5 min at 4 °C. The pellets were resuspended in 10 mL of sterile ultrapure water and counted using a hemocytometer. The conidia were inoculated into a yeast extract-peptone (YEP) broth supplemented with APAP and divided into 50 mL flasks, incubated for two weeks at 25 ± 2 °C with constant shaking at 126 rpm using an IKA-WERKE shaker (IKA-Werke, Staufen, Germany) [33].

#### 2.7.3. Bioanode Preparation

A 10 cm^2^ piece of carbon felt (CF) from Carbon Lorraine, France was used as the fungal biofouling support. The CF was sequentially cleaned with 1 M HCl, ultrapure water, and a 1:1 ethanol/water solution, followed by sonication in ultrapure water (2 min at 47 kHz) for ethanol rinsing. The CF was then autoclaved at 120 °C for 15 min. It was colonized with *Scedosporium dehoogii* spores by immersion in a spore suspension for one week. The biofilm was developed by polarizing the CF at 0.15 V vs. SCE (saturated calomel electrode—reference electrode) for one week under sterile conditions, using 4-hydroxybenzoate (4 HBz) as the carbon source at 0.9 g/L [32].

#### 2.7.4. Cathode Preparation

The CF cathode underwent electrochemical treatment through cyclic voltammetry (CV) between 0.0 and 1.2 V vs. SCE in a 0.1 M NaOH solution for 10 cycles. Optimal electrodeposition had been achieved at a potential of 0.8 V vs. SCE [32] (Pontié et al., 2019). Poly-Ni(II)tetrasulphonated phthalocyanine (Poly-NiTSPc) was then electrochemically deposited on the cathode in a solution of 0.1 M NaOH and 2 mM Ni(II)tetrasulphonated phthalocyanine (NiTSPc) at +0.8 V vs. SCE for 3000 s. During the initial scan for oxidative potential at E = 0.8 V, OH° is formed via water oxidation, creating various functional groups on the electrode surface. These groups facilitate the coupling of NiTSPc complexes to the surface through –O–Ni(II) bonds (oxo-bridges). With continued oxidation, Ni(II) complexes transform into Ni(III) and bond with new Ni-complexes via oxo-bridges. As a result, the film structure is imposed by the stacking of the complex layers via interconnecting oxo-bridges. In the microbial fuel cell (MFC) setup, the cathode acts as an electrode where the electron acceptors are reduced and typically undergoes oxygen reduction. In the double-chamber MFC, oxygen (the electron acceptor in the cathodic chamber) is continually sparged into the cathodic solution from ambient air [34]. To minimize bacterial contamination, a 0.45 μm air filter (Biblock, Paris, France) was used to filter the incoming air in the cathodic compartment [34].

#### 2.7.5. MFC Set-Up

A dual-chamber configuration was adopted for this study, with the anodic and cathodic compartments separated by a membrane that allows protons to move through it. On the anodic side, the solution consists of 100 mg L^−1^ APAP (Acetaminophen) in a phosphate buffer solution (PBS) 0.1 M, pH 7.4, while the cathodic solution consists of 0.1 M PBS at the same pH. Oxygen from an air pump was supplied to the cathode chamber and filtered through a 0.45 μm filter. An external resistance of 1 kΩ connected the electrodes to facilitate electron flow from the anode to the cathode [35,36]. Power output was measured using this external resistance during MFC discharge. Electrons generated through the fungal oxidation of the APAP substrate were directly transferred to the anode, then flowed to the cathode through a conductive material containing a resistor. The electrons that reach the cathode combine with protons diffused through the membrane from the anode and with oxygen from the air, resulting in water production. This electron flow from the anode to the cathode generates the necessary current and voltage to produce electricity.

The resistance of the membrane was determined using Ohm’s law (Equation (4)):

*U* = *R*·*I* (4)

where *U* represents the electromotive force, *R* is the resistance (Ohm), and *I* is the current intensity (A).

Power densities are calculated using Equation (5):

*P* = *U*·*I* (5)

where *U* is the electromotive force and *I* is the current intensity.

To collect data on voltage, current, and power, external resistances ranging from 10 MOhm to 10 Ohm were applied through a resistance box. Power and current values were then normalized to the electrode surface area to calculate power and current densities.

### 2.8. LCA Studies

Life cycle assessment (LCA) provides a method to evaluate the potential environmental impact of a system throughout its life cycle. With growing interest in assessing the sustainability of membrane treatment systems, particularly for wastewater treatment (WWT), LCA studies contribute to exploring innovative uses for end-of-life reverse osmosis (EoL-RO) membranes as functional components in MFCs. This dimension of analysis is crucial to understanding the environmental viability of these technologies. While the use of chlorine (Cl_2_) for membrane transformation is a viable option, its environmental impact merits scrutiny. To take this a step further, we provide LCA results of chlorine treatment on the membranes and explore alternative treatment options. In Table 1, input/output data for proposed scenarios for LCA studies are presented. Cutoff refers to the allocation method used in the Ecoinvent database, which distributes environmental impacts based on the economic value of co-products. U (Unit process) indicates that the data represent individual processes, ensuring traceability and specificity in the LCA model.

Open LCA software (version 2.0.4) and the Ecoinvent 3.4 cut-off database (access provided via regional funding dispositive titled Pulsar with convention number of 2022_09582) were used to analyze and compare treatment options. To isolate the impact of the choice of treatment solution, a functional unit of 0.01 m^2^ of the EoL RO membrane module was established. The ReCiPe Midpoint (E) method from the Ecoinvent v.3.3 LCIA methods was chosen to study different impact categories.

## 3. Results and Discussion

### 3.1. Investigation of Membrane Performance

#### 3.1.1. Hydraulic Permeability

**Figure 1:**
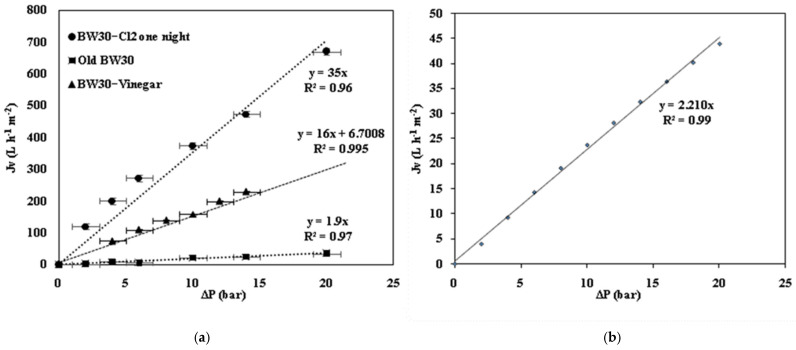
Effect of operating pressure on the pure water permeate flux J_v_ for (**a**) the EoL RO membrane, the EoL RO membrane after one night in chlorine (Cl_2_), and the EoL RO membrane after one night in vinegar; and (**b**) simulation results of the BW30-400 at 20 °C.

The effect of operating pressure on the pure water permeate flux (J_v_) for the different studied membranes, along with simulation results for BW30-400 at 20 °C, is shown in Figure 1. Initially, the time taken to collect a constant volume (20 mL) of permeate at various pressures (2–20 bar) was measured after the membranes were placed in the module. Figure 1 presents the permeate flux, calculated from the collected flow rate volume, as a function of pressure.

According to Equation (1), there is a linear relationship between the permeate flux (J_v_) and the applied transmembrane pressure. At 20 °C, the hydraulic permeability of the EoL RO membrane was 1.9 L·h^−1^·m^−2^·bar^−1^, a reduction from the reference membrane, indicating it has undergone fouling and no longer exhibits the same permeability as a new RO membrane.

For a known temperature (20 °C) and fora well-defined UF membrane (PS UF 100 kDa) with a hydraulic permeability of *L_p_*_0_ = 100 L·h^−1^·m^−2^·bar^−1^ and *d_p_*_0_ = 7.6 nm, we can determine the average pore diameter (*d_p_*) using Equation (6) and applying the known *L_p_* = 35 L h^−1^·m^−2^·bar^−1^ [37]:

*L*_*p*_/*L*_*p*0_ = (*d*_*p*_/*d*_*p*0_)^2^ (6)

The *d_p_* obtained is 6 nm from the previous Equation (6).

Using the *d_p_* value, the molecular weight cut-off (*MWCO*) of the membranes treated with chlorine and vinegar can be estimated using Equation (7).

*d*_*p*_ = 0.076·(*MWCO*)^0.4^ (7)

where the *d_p_* is in nm and *MWCO*, the molecular weight cut-off, is in Da.

For chlorine treatment, a *MWCO* of 26,977 Da was obtained, classifying it as an ultrafiltration membrane. In the case of the *L_p_*_0_ = 16, we obtained a *MWCO* of 9789 Da.

#### 3.1.2. Autopsy Experiments

**Figure 2:**
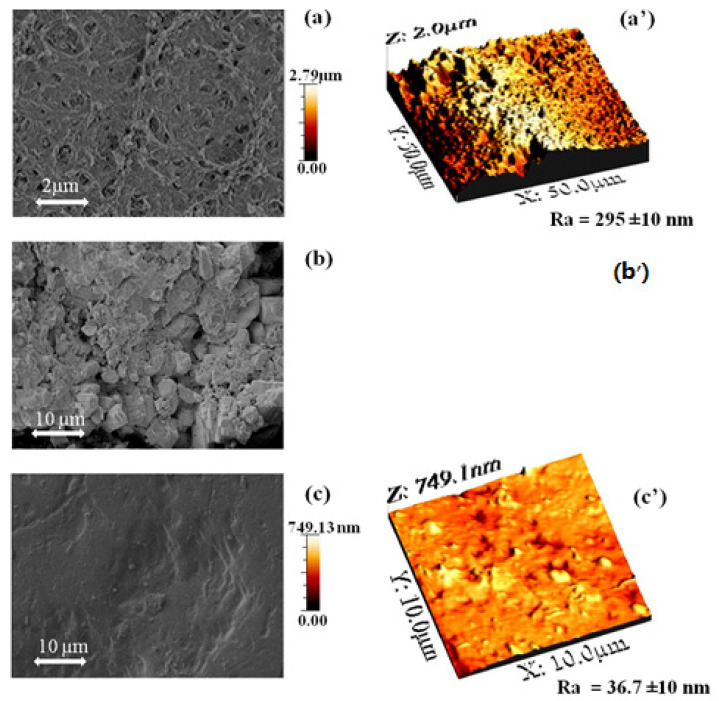
(**a**,**a′**) SEM/AFM images of old pristine RO membrane [16], (**c**,**c′**) the EoL RO membrane after one night in chlorine (Cl_2_) and (**b**) SEM image of the EoL RO membrane and (**b′**) not determined (the roughness was too high to be determined in AFM).

Figure 2 presents SEM and 3D-AFM images of the old pristine BW30 membrane, the EoL BW30 membrane, and the EoL BW30 membrane after one night in Cl_2_. The SEM morphologies in Figure 2a–c, reveal visible differences in the previous membranes surface morphology, demonstrating the effect of Cl_2_ oxidation on the PA layer.

In a previous study [16], AFM analysis of old pristine RO membrane showed an average roughness of 295 nm over a 50 × 50 µm^2^ area. Following Cl_2_ treatment for one night, the membrane surface became more homogeneous and smoother (see Figure 2c), effectively converting an EoL BW30 membrane into a new UF membrane with an MWCO of approximately 55 kDa. Various optical techniques have been reported in the literature for measuring surface roughness. Optical microscopy, used for general assessment, only provides qualitative information [38].

**Figure 3:**
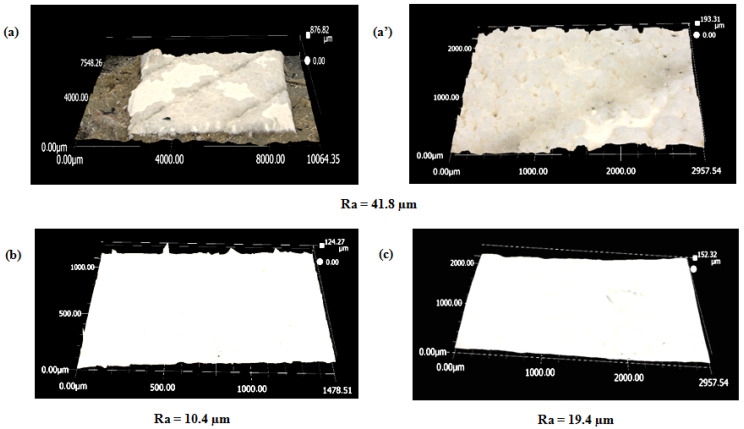
Roughness analysis using Keyence optical microscopy of (**a**,**a′**) the EoL RO membrane, (**b**) the EoL RO membrane after one night in vinegar, and (**c**) the EoL RO membrane after one night in chlorine (Cl_2_).

In Figure 3, optical microscopy reveals roughness analyses of the EoL RO membrane surface after being soaked for one night in vinegar and one night in chlorine (Cl_2_). Following these treatments, the membrane surface became more homogeneous and smoother (see Figure 3b,c). The estimated roughness values (Ra) for the vinegar and Cl_2_ treatments were 10.4 µm and 19.4 µm, respectively, compared to the EoL RO membrane’s *Ra* of 41.8 µm (see Figure 3a,a′). This reduction in *Ra* indicates that both the vinegar and Cl_2_ altered the membrane surface texture.

**Figure 4:**
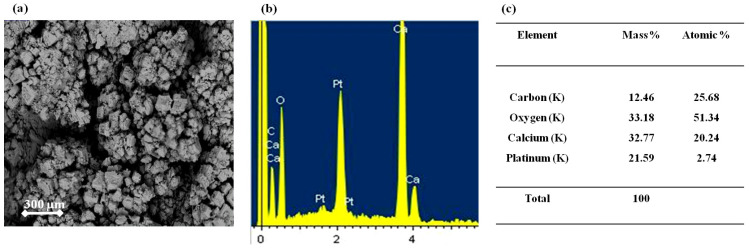
(**a**) SEM image of crystals coating the surface of the EoL RO membrane and (**b**,**c**) elemental analysis of these crystals.

As shown in Figure 4a, a compact layer of salt crystals is visible on the membrane surface. Energy dispersive X-ray spectroscopy (EDS) analysis was performed to identify the composition of these crystals and estimate their proportion, as presented in Figure 4b,c. This analysis detected the presence of the following elements: Pt, Ca, O, and C. The Pt originates from a conductive coating applied during microscopy to enhance the quality of the images. Atomic percentages for the other elements are reported in Figure 4c.

The data reveal an atomic percentage ratio of Ca/O of around 2.6, or close to 3, suggesting that the deposition is CaCO3. This hypothesis is further supported by the rhombohedral shape of the crystals which is characteristic of calcite.

**Figure 5:**
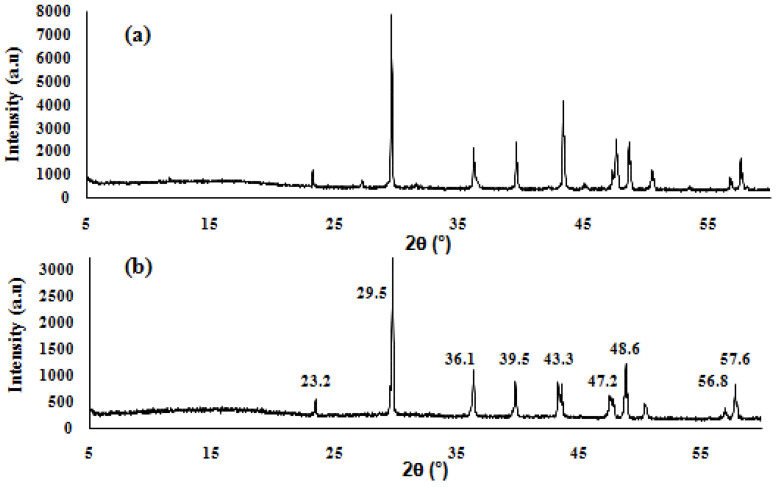
X-ray diffractograms of the (**a**) crystals coating the surface of the EoL RO membrane and (**b**) calcite reference.

To confirm that the deposition is indeed CaCO_3_ on the surface of the EoL RO membrane, an XRD analysis was conducted. Figure 5 presents the X-ray diffraction of the crystals coating the surface of the EoL RO membrane, showing the presence of narrow peaks that correspond to the diffraction peaks of CaCO_3_ (reference). The diffraction peaks at 29.5°, 36.1°, and 39.5°correspond to the (1 0 4), (1 1 0), and (1 3 3) of calcite, respectively [39,40]. Notably, the intense peak at 2θ = 29.5° suggests that calcite is the only form of calcium carbonate solids formed on the surface of the EoL RO membrane [41].

**Figure 6:**
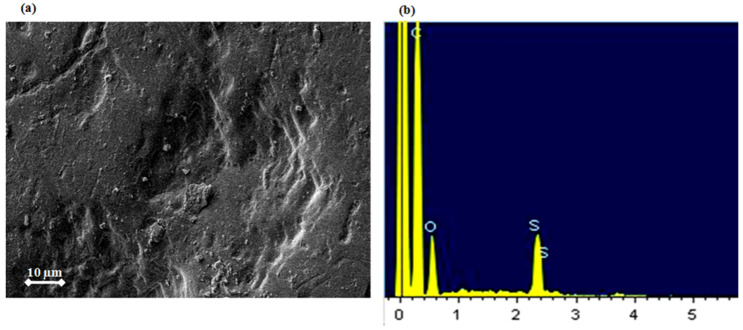
(**a**) SEM image and (**b**) chemical analysis by energy dispersive X-ray spectrometry at 20 keVofEoL RO membrane after one night in chlorine (Cl_2_).

SEM images and EDX spectra of the aged BW30 membrane after one night of exposure to Cl_2_ are shown in Figure 6. As seen in the SEM morphologies (Figure 6a), the membrane surface became more homogeneous and smoother. The effects of the Cl_2_ oxidation on the PA layer are illustrated in Figure 6b. The presence of sulfur in the spectrum comes from the polysulfone support layer, while the absence of calcium demonstrates the near-complete removal of the CaCO_3_ layer.

### 3.2. Deposition of Amer Sil on Membrane Surface After Transformation by Cl_2_

**Figure 7:**
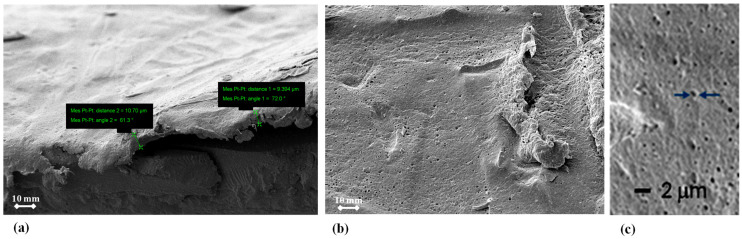
(**a**) SEM image showing the thickness of the cationic exchange active layer surface, (**b**) SEM image of the surface at ×1000 magnification, and (**c**) close-up at ×5 magnification, showing a zone of observed holes.

SEM images at ×1000 magnification of the cationic exchange active layer deposited on the membrane surface are presented in Figure 7. The SEM analysis confirms the deposition of this active layer, which is composed of an interpenetrating polymer network (IPN) made of sulfonated polyether sulfone (S-PES) in a crosslinked matrix of acrylic acid and divinylbenzene.

**Figure 8:**
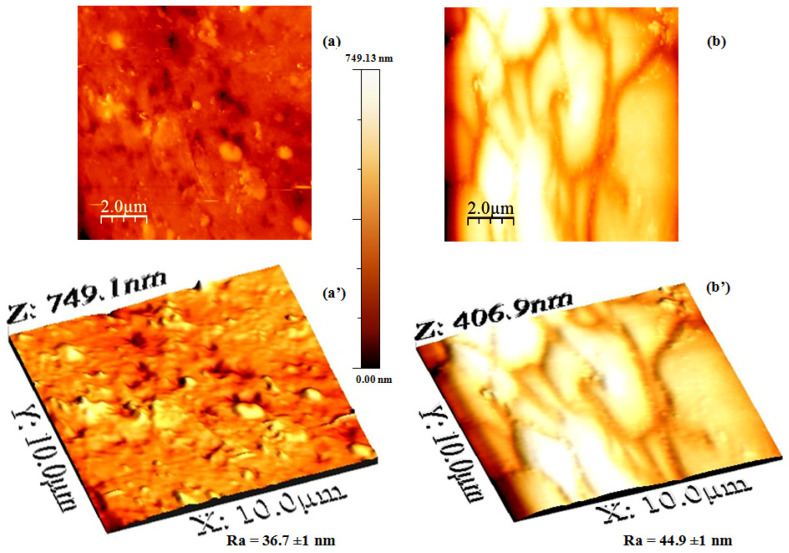
2D and 3D topographical images of (**a**,**a′**) the EoL RO membrane after soaking for one night in chlorine (Cl_2_) and (**b**,**b′**) the EoL RO membrane after soaking for one night in chlorine (Cl_2_) and with Amer Sil deposition.

2D and 3D-AFM images of the EoL RO membrane treated with chlorine (Cl_2_), both with and without the Amer Sil deposit, are presented in Figure 8. When Amer Sil is present, the roughness (Ra) is estimated at 44.9 ± 1 nm, compared to 36.7 ± 1 nm for the membrane treated only with Cl_2_. This slight decrease in Ra demonstrates that the PSS has changed the characteristics of the membrane surface.

### 3.3. FTIR Results

**Figure 9:**
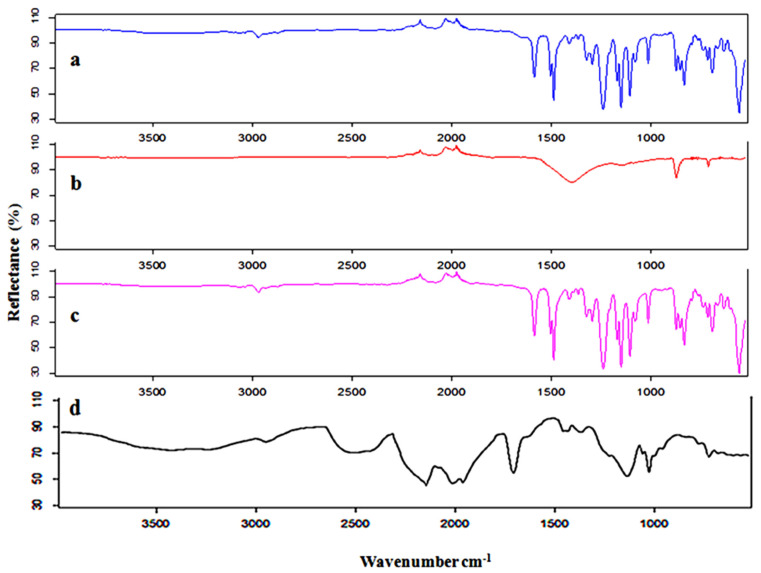
FTIR spectra are shown for (**a**) the old pristine RO membrane, (**b**) the EoL RO membrane, (**c**) the EoL RO membrane after one night in chlorine (Cl_2_), and (**d**) the EoL RO membrane after one night in chlorine (Cl_2_) with Amer Silde position.

The functional groups and chemical structure of the old pristine RO membranes, after Cl_2_ treatment and with Amer Sil deposition, are analyzed by FTIR spectroscopy as shown in Figure 9. The spectrum of the EoL RO membrane (b) was very different than the spectrum of the EoL RO membrane after one night in chlorine (Cl_2_) (c). The characteristic peaks of CaCO_3_ at 1391.2 cm^−1^, 867.6 cm^−1^, and 706.2 cm^−1^ appear only in spectrum (b) [42], confirming that the CaCO_3_ layer has been completely removed after Cl_2_ treatment. The FTIR spectrum of the PA layer exhibits characteristic functional groups at 1583 cm^−1^ corresponding to the amide II band associated with N-H plane bending. This result is consistent with that observed in other studies [16]. By comparing the spectrum of the old pristine RO membrane (a) and the EoL RO membrane after one night in chlorine (Cl_2_) (c), it can be seen that the intensity of polyamide peaks is reduced with the Cl_2_ exposure intensity.

In Figure 9d, the peak of the –SO_3_^−^ symmetric stretching vibration shifts slightly from 1056 to 1060 cm^−1^, indicating interactions between the water molecules and the sulphonic group. The peaks at 1135 and 1204 cm^−1^ may be assigned to the –SO_3_^−^ group asymmetric stretching vibration [43].

### 3.4. Applications of Modified End-of-Life RO Membranes

#### 3.4.1. Domestic Effluent Treatment

Artificial domestic effluent was prepared using particles (MICROCOL ALPHA) a at approximately 50.3 NTU per 0.2 g of clay per liter of tap water, combined with3-methyl-4-nitrophenol (MNP) and tap water. This compound is an organic micro-pollutant and the main by-product of fenitrothion, an organophosphorus insecticide used in the Sahel to combat invasions of the pilgrim cricket.

**Figure 10:**
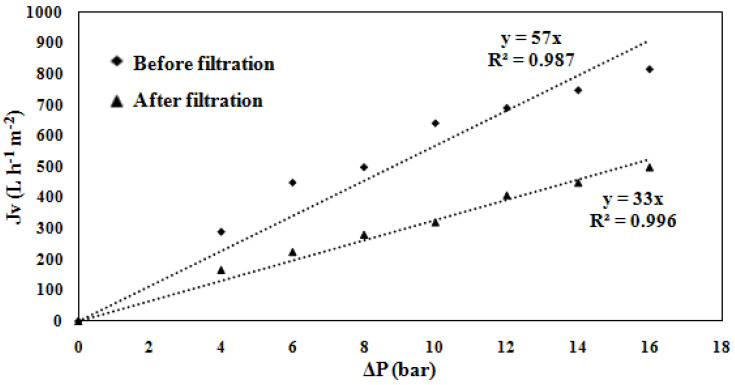
Evolution of the flux *J_v_* before and after filtration of artificial domestic effluent as a function of pressure for the prepared UF membrane.

After filtering the artificial domestic effluent, the membrane’s permeability measured 33 L·h^−1^·m^−2^·bar^−1^ (Figure 10), reflecting a 42% decrease, indicating that the synthetic solution of clay and MNP has clogged the membrane. A cleaning step may be carried out to eliminate this fouling.

**Figure 11:**
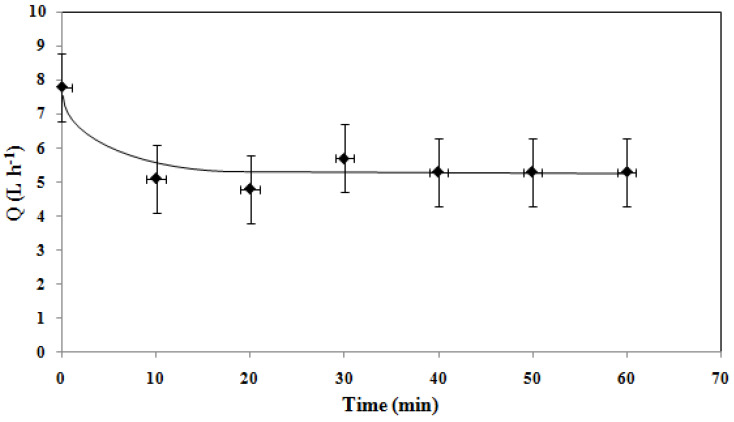
Evolution of permeate flow overtime during the filtration of artificial domestic effluent (Δ*P* = 6 bar).

As shown in Figure 11, the flow decreases in the first 10 min and then stabilizes at 5 L·h^−1^. The stabilized flux can be calculated using Equation (8).

*J*_*stab*_ = *Q*_*stab*_/*S* (8)

where *J_stab_* is the stabilized flux, *Q_stab_* is the stabilized flow, and *S* is the membrane surface (200 cm^2^).

Furthermore, with a *J_stab_* value of 250 L·h^−1^·m^−2^, we can estimate what surface of the membrane of 40 m^2^ is needed to produce a flow of 10,000 L·h^−1^ at 6 bar. For this purpose, a commercial 8-inch membrane of 37 m^2^ should be used.

**Figure 12:**
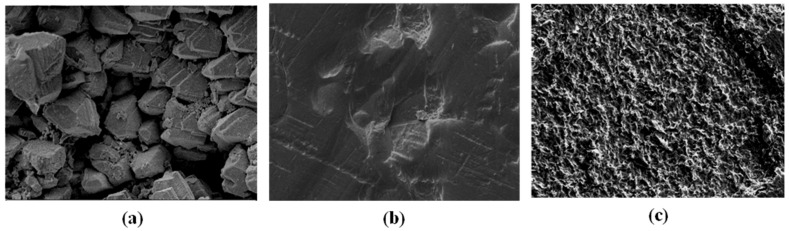
SEM images of membrane surfaces (**a**) before acetic acid treatment, (**b**) following acetic acid treatment, and (**c**) following artificial domestic effluent filtration.

To illustrate the decrease in permeate flow over time, Figure 12 shows how the clay particles accumulate on the new membrane, resulting in clogging. Our experiment focused on monitoring turbidity, conductivity, and MNP concentration during the artificial domestic effluent filtration at 6 bars, summarized in Table 2.

While conductivity and MNP concentration remains nearly unchanged during filtration, turbidity dropped significantly from 50.2 NTU tonearly zero (<0.1 NTU), indicating that this new UF membrane has a high level of efficiency in removing5 µm bentonite particles.

#### 3.4.2. FMFC Performance

**Figure 13:**
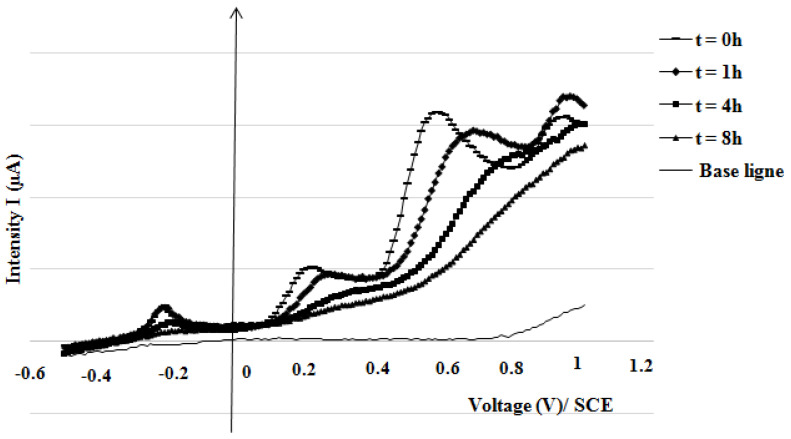
Square-wave voltammetry curves of micropollutant mixtures over time on the p-NiTSPc-CFME.

Figure 13 shows square-wave voltammetry (SWV) curves of micropollutant mixtures over time after inoculating the liquid medium with *Scedosporium dehoogii.* The fungus’ sconsumption of methylene blue (MB), para-aminophenol (PAP), paracetamol (APAP), and Sulfanilamide (SFA) was monitored using our custom carbon paste electrodes (CPE). Peaks for each micropollutant disappeared within 8 h, showing that *Scedosporium dehoogii* biodegrades these micropollutants by converting them into substrates that are more easily metabolized.

**Figure 14:**
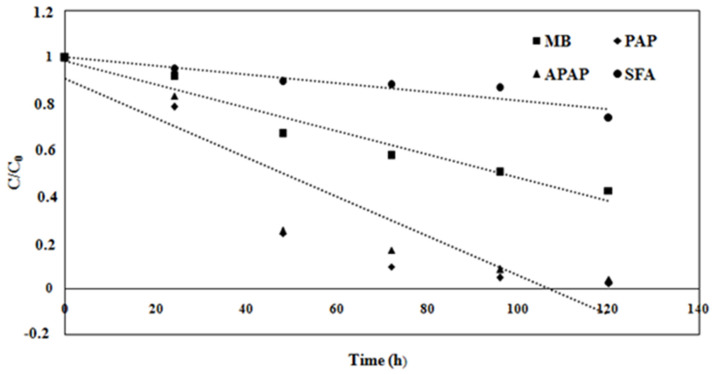
Biodegradation kinetics of MB, PAP, APAP, and SFA by *S. dehoogii* at 20 °C.

In Figure 14, plots of C/C0 as a function of time indicate that micropollutant concentrations steadily decreased as the fungal malt in the flasks decreased. This result supports the hypothesis that the fungus biodegrades the micropollutants and uses them as a carbon source.

The kinetic reaction follows a pseudo-first-order process, with constants and half-life values shown in Table 3. The results for PAP biodegradation are superior to those of APAP from previous studies using the same FMFC set-up [32], where the kinetic constant and half-life were 0.19 day^−1^ and 3.3 days, respectively.

**Figure 15:**
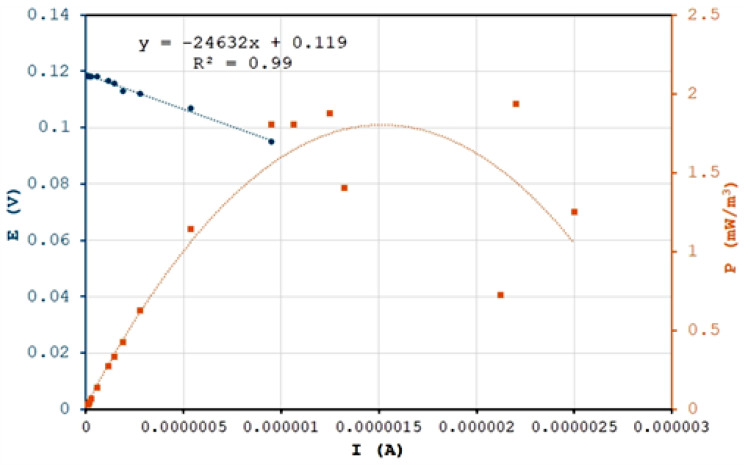
Polarization curve for the modified EoL RO membranein the MFC.

To assess the modified membrane’s suitability as a PEM in an FMFC, we tested the modified EoL RO membrane in the reactor. Figure 15 shows the polarization curve of the modified membrane in the microbial fuel cell, plotting voltage, current, and power, which are critical metrics for evaluating MFC performance. We observe that, regardless of voltage, the modified EoL RO membrane reached a power density of 2 mW/m^2^. This may be due to the membrane’s internal resistance, calculated from the voltage-to-current density slope to be approximately 25,000 Ohm. Comparatively, this membrane is more conductive than the Nafion^®^ 117 membrane, which has an internal resistance of 8000 Ohm [16]. Lower resistance minimizes energy loss, contributing to acceptable power density. At maximum power density, the modified EoL RO membrane reached a current of 1.6 µA.

### 3.5. LCA Studies

**Figure 16:**
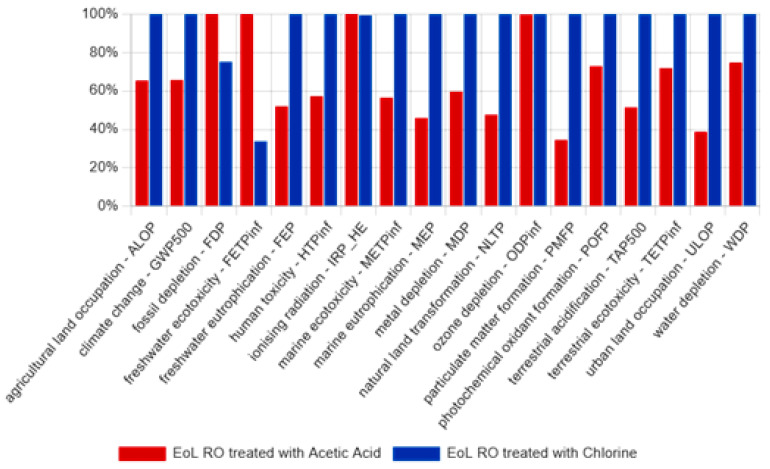
LCIA results comparing acetic acid and chlorine treatments for EoL RO membrane cleaning.

LCA offers a systematic framework for evaluating the environmental impacts of a product’s life cycle, from raw material extraction to end-of-life disposal. In this context, alternative membrane treatments, such as exposure to acetic acid, appear to offer promising ways of reducing environmental harm. Acetic acid, a milder and more environmentally friendly agent than chlorine, offers an effective alternative for membrane modification. Figure 16 presents life cycle impact assessment (LCIA) results for each ILCD category comparing chlorine and acetic acid treatments. For each indicator, the maximum result is set to 100%, and the other variants’ results are presented relative to this benchmark.

Detailed comparisons reveal that acetic acid treatment of EoL RO membranes has lower environmental impacts than chlorine in several categories, including agricultural land use (0.00019 m^2^a vs. 0.00030 m^2^a), climate change (0.73377 kg CO_2_-Eq vs. 1.11673 kg CO_2_-Eq), freshwater eutrophication (0.00023 kg P-Eq vs. 0.00044 kg P-Eq), human toxicity (10.37395 kg 1,4-DCB-Eq vs. 18.10529 kg 1,4-DCB-Eq), and marine ecotoxicity (8.86732 kg 1,4-DCB-Eq vs. 15.69255 kg 1,4-DCB-Eq). However, chlorine has performed better in terms of fossil depletion (0.22049 kg oil-Eq vs. 0.29325 kg oil-Eq) and freshwater ecotoxicity (0.01855 kg 1,4-DCB-Eq vs. 0.05488 kg 1,4-DCB-Eq). These reductions are primarily due to the less harmful byproducts and lower toxicity associated with acetic acid, resulting inreduced environmental degradation and better health outcomes for ecosystems. While the two treatments have similar effects on ionizing radiation, the overall data suggest that acetic acid is the more sustainable choice for minimizing environmental impacts.

The environmental benefits of repurposing EoL RO membranes align with findings from previous studies. For example, Lawler et al. [12] demonstrated that recycling RO membranes reduces greenhouse gas emissions compared to landfill disposal or incineration. Similarly, Senán-Salinas et al. [2] emphasized the role of recycling in minimizing the climate change impact of desalination processes. These studies support the trends observed in our results, which highlight the potential of acetic acid treatment as a more sustainable alternative to chlorine.

While chlorine treatment remains a widely used method due to its efficiency in removing fouling and modifying membrane properties [28], its environmental trade-offs are evident. The increased marine ecotoxicity and freshwater eutrophication impacts identified in this study are consistent with findings by Lejarazu-Larrañaga et al. [29], who reported similar trends in the recycling of thin-film composite membranes. On the other hand, acetic acid treatment shows promise as a milder, less harmful option, as suggested by Rehman et al. [6], who noted its compatibility with circular economy approaches.

It is important to address the uncertainties and limitations inherent in LCA studies to provide a more robust interpretation of these results. The variability of input data in the Ecoinvent database, particularly for electricity and chemical inputs, can influence the outcomes. Additionally, the study’s boundaries are limited to the immediate membrane treatment process, excluding upstream processes like chemical production and downstream impacts post-treatment. The selection of the ReCiPe Midpoint (E) impact assessment method may also introduce variability, as different LCIA methods could yield different category-wise impacts. These factors highlight the need for cautious interpretation of the results while acknowledging the overall trends observed in the study. To enhance the robustness of the findings, future research should focus on integrating sensitivity analyses and life cycle cost assessments to provide a more holistic understanding of the trade-offs. Additionally, exploring hybrid treatment methods that combine the strengths of chlorine and acetic acid could further optimize both environmental and functional outcomes.

## 4. Conclusions

This research explores the innovative dual repurposing of end-of-life BWRO membranes, transforming them into ultrafiltration membranes for advanced wastewater treatment and cation exchange membranes for fungal microbial fuel cells.

The EoL RO membranes exhibited significant degradation due to fouling and chemical exposure during their initial life cycle in water softening applications. The analysis of water permeability, roughness, and chemical composition revealed the extent of this degradation. The application of acetic acid treatment effectively transformed the EoLRO membranes into functional UF membranes suitable for domestic effluent treatment, demonstrating increased permeability comparable to standard UF membranes. Chlorine treatment followed by the deposition of a sulfonated layer enhanced the membranes’ performance for use in fungal microbial fuel cells (FMFC), indicating their viability in energy generation applications. Advanced analytical methods, including scanning electron microscopy (SEM), energy dispersion spectroscopy (EDS), and Fourier transform infrared spectroscopy (FTIR), confirmed the effectiveness of the treatments. The LCA highlighted the environmental benefits of recycling EoL membranes, although it also pointed out the uncertainties and limitations inherent in such assessments. Future studies should investigate the long-term stability and performance of the repurposed membranes in real-world applications, such as wastewater treatment and microbial fuel cells, which will provide valuable insights into their practical viability.

## Figures and Tables

**Table 1:** Input/output data for proposed scenarios in LCA studies.

	Flow	Unit	Provider
S1: EoL RO treated with Acetic Acid			
Input	acetic acid, no water, in 98% solution	kg	acetic acid production, product in 98% solution state|acetic acid, without water, in 98% solution state|Cutoff, U
	electricity, low voltage	kWh	market for electricity, low voltage|electricity, low voltage|Cutoff, U
	seawater reverse osmosis module	m^2^	seawater reverse osmosis module production, 8-inch spiral wound, enhanced|seawater reverse osmosis module|Cutoff, U
	water, deionized, from tap water, at user	kg	water production, deionized, from tap water, at user|water, deionized, from tap water, at user|Cutoff, U
	water, ultrapure	kg	market for water, ultrapure|water, ultrapure|Cutoff, U
Output	EoL RO treated with Acetic Acid	m^2^	
	acetic acid	kg	
	heat, waste	kWh	
	water, waste	kg	
S2: EoL RO treated with Chlorine			
Input	electricity, low voltage	kWh	market for electricity, low voltage|electricity, low voltage|Cutoff, U
	seawater reverse osmosis module	m^2^	seawater reverse osmosis module production, 8-inch spiral wound, enhanced|seawater reverse osmosis module|Cutoff, U
	sodium hypochlorite, no water, in 15% solution	kg	market for sodium hypochlorite, no water, in 15% solution|sodium hypochlorite, no water, in 15% solution|Cutoff, U
	water, ultrapure	kg	market for water, ultrapure|water, ultrapure|Cutoff, U
Output	EoL RO treated with Chlorine	m^2^	
	heat, waste	kWh	
	sodium hypochlorite	kg	
	water, waste	kg	

**Table 2:** Changes in conductivity, turbidity, and MNP concentration during artificial domestic effluent filtration at 6 bars (initial conditions: conductivity = 605 μS/cm, turbidity = 50.2 NTU, and MNP concentration = 500 mg/L).

Time (min)	Conductivity (µS/cm)	Turbidity (NTU)	MNP (mg/L)
0	605	<0.1	500
10	573	<0.1	462
20	588	0.17	612
30	590	0.24	583
40	591	0.15	525
50	576	<0.1	475

**Table 3:** Kinetic constants, half-lives, and R^2^ values of MB, PAP, APAP, and SFA.

	MB	PAP	APAP	SFA
Kinetic constant k (h^−1^)	0.0037	0.0311	0.0260	0.0020
Half-life t_1/2_ (h)	94.9516	22.2876	26.6595	301.3683
R^2^	0.9790	0.9760	0.9720	0.9540

## Data Availability

The data presented in this study are available on request from the corresponding author.
